# Reshaping the path of vascular cognitive impairment with resistance training: a study protocol for a randomized controlled trial

**DOI:** 10.1186/s13063-021-05156-1

**Published:** 2021-03-18

**Authors:** Teresa Liu-Ambrose, Elizabeth Dao, Rachel A. Crockett, Cindy K. Barha, Ryan S. Falck, John R. Best, Ging-Yeuk R. Hsiung, Thalia S. Field, Kenneth M. Madden, Walid A. Alkeridy, Narlon C. Boa Sorte Silva, Jennifer C. Davis, Lisanne F. ten Brinke, Stephanie Doherty, Roger C. Tam

**Affiliations:** 1 Department of Physical Therapy, University of British Columbia (UBC), Vancouver, British Columbia Canada; 2 Djavad Mowafaghian Centre for Brain Health, Vancouver, British Columbia Canada; 3 Centre for Hip Health and Mobility, Vancouver Coastal Health Research Institute, Vancouver, British Columbia Canada; 4 Department of Radiology, UBC, Vancouver, British Columbia Canada; 5 International Collaboration on Repair Discoveries, Vancouver, British Columbia Canada; 6 Gerontology Research Centre, Simon Fraser University, Vancouver, British Columbia Canada; 7 Department of Gerontology, Simon Fraser University, Vancouver, British Columbia Canada; 8 Department of Psychiatry, UBC, Vancouver, British Columbia Canada; 9 Division of Neurology, UBC, Vancouver, British Columbia Canada; 10 Vancouver Stroke Program, Vancouver, British Columbia Canada; 11 Division of Geriatric Medicine, Department of Medicine, UBC, Vancouver, British Columbia Canada; 12 Department of Medicine, College of Medicine, King Saud University, Riyadh, Saudi Arabia; 13 Social and Economic Change Laboratory, Faculty of Management, UBC–Okanagan, Kelowna, British Columbia Canada; 14 School of Biomedical Engineering, UBC, Vancouver, British Columbia Canada

**Keywords:** Randomized controlled trial, Vascular cognitive impairment, Resistance training, Cognitive Function, Mobility, Exercise, White matter hyperintensities

## Abstract

**Background:**

Subcortical ischemic vascular cognitive impairment (SIVCI) is the most common form of vascular cognitive impairment. Importantly, SIVCI is considered the most treatable form of cognitive impairment in older adults, due to its modifiable risk factors such as hypertension, diabetes mellitus, and hypercholesterolemia. Exercise training is a promising intervention to delay the progression of SIVCI, as it actively targets these cardiometabolic risk factors. Despite the demonstrated benefits of resistance training on cognitive function and emerging evidence suggesting resistance training may reduce the progression of white matter hyperintensities (WMHs), research on SIVCI has predominantly focused on the use of aerobic exercise. Thus, the primary aim of this proof-of-concept randomized controlled trial is to investigate the efficacy of a 12-month, twice-weekly progressive resistance training program on cognitive function and WMH progression in adults with SIVCI. We will also assess the efficiency of the intervention.

**Methods:**

Eighty-eight community-dwelling adults, aged > 55 years, with SIVCI from metropolitan Vancouver will be recruited to participate in this study. SIVCI will be determined by the presence of cognitive impairment (Montreal Cognitive Assessment < 26) and cerebral small vessel disease using computed tomography or magnetic resonance imaging. Participants will be randomly allocated to a twice-weekly exercise program of (1) progressive resistance training or (2) balance and tone training (i.e., active control). The primary outcomes are cognitive function measured by the Alzheimer’s Disease Assessment Scale-Cognitive-Plus (ADAS-Cog-13 with additional cognitive tests) and WMH progression.

**Discussion:**

The burden of SIVCI is immense, and to our knowledge, this will be the first study to quantify the effect of progressive resistance training on cognitive function and WMH progression among adults with SIVCI. Slowing the rate of cognitive decline and WMH progression could preserve functional independence and quality of life. This could lead to reduced health care costs and avoidance of early institutional care.

**Trial registration:**

ClinicalTrials.gov NCT02669394. Registered on February 1, 2016

**Supplementary Information:**

The online version contains supplementary material available at 10.1186/s13063-021-05156-1.

## Introduction

Worldwide, one new case of dementia is detected every 4 s [1]. Cerebrovascular disease, such as stroke, is the second most common cause of dementia after Alzheimer’s disease (AD) [2–5], accounting for up to 38% of all dementia cases [6]. Much of stroke research to date has focused on overt ischemic strokes. However, covert ischemic strokes may outnumber overt strokes by five to one, and approximately 25% of people over 80 years of age have one or more silent brain infarcts [7]. Consequently, the prevention of covert ischemic stroke is now a recognized research priority [8].

Vascular cognitive impairment (VCI) encompasses all levels of cognitive decline, from mild cognitive deficits to dementia, due to both overt and covert cerebrovascular disease [9]. The most common cause of VCI is cerebral small vessel disease, in which covert ischemic damage to the brain leads to the development of subcortical ischemic vascular cognitive impairment (SIVCI) [10–12]. In SIVCI, cerebrovascular damage predominantly manifests as white matter hyperintensities (WMHs) of presumed vascular origin and lacunes. Symptoms include prominent impairment in processing speed and executive functions, but can also include impaired memory, language, and visuospatial functions [13, 14]. Functional impairments such as gait disturbance, unsteadiness, and frequent, unprovoked falls are also present [15]. Overall, the clinical consequences of covert ischemic strokes are substantial.

Fortunately, SIVCI may be the most treatable form of cognitive dysfunction in older adults because its key risk factors, which include hypertension, diabetes mellitus, and hypercholesterolemia, are modifiable. Exercise is a promising approach to delay the progression of SIVCI [16–20], as it can effectively modify key cardiometabolic risk factors [16–20], improve vascular function, and alter response during ischemia [21, 22]. Middleton and colleagues [23] demonstrated in the Canadian Study of Health and Aging cohort that physical activity reduced the risk of SIVCI. A cross-sectional study of 1238 people with no history of overt stroke found that those in the highest quartile of physical activity were almost 50% less likely to present evidence of covert stroke compared to those who reported no regular weekly exercise [24].

However, the best type of exercise intervention for persons with SIVCI is unknown. Broadly, the two most common forms of exercise training are (1) aerobic training (e.g., running) and (2) resistance training (e.g., lifting weights). Current research efforts in SIVCI and exercise focus primarily on aerobic training [25, 26] despite evidence that suggests resistance training has important benefits for cardiometabolic health [27–30], cognitive function [31–34], and notably, WMH progression [35, 36]. Resistance training may also benefit individuals with SIVCI by directly moderating muscle loss (i.e., sarcopenia) whereas aerobic exercise does not. Sarcopenia is a risk factor for impaired mobility and falls [37], which are associated with WMHs [15, 38, 39]. Preliminary evidence suggests that resistance training may be beneficial for people with SIVCI. A 12-month randomized controlled trial (RCT) with community-dwelling older women showed progressive resistance training (PRT) significantly improved executive functions [32] and slowed WMH progression [35], compared with an active control group [35].

The mechanisms by which resistance training may promote cognitive function are not well established. One potential mechanism is through the upregulation of neurotrophic factors such as brain-derived neurotrophic factor (BDNF), vascular endothelial-derived growth factor (VEGF), and insulin-like growth factor-1 (IGF-1) [40, 41]. These neurotrophic factors are thought to mediate the beneficial effects of exercise on brain plasticity and cognitive function [40, 41]. Notably, resistance training is especially effective for increasing levels of serum IGF-1 in older adults [33]. IGF-1 in the periphery can pass through the blood–brain barrier where it is involved in vascular maintenance and remodeling [42]—reductions in IGF-1 are associated with decreased cerebral vascular density and blood flow [43]. IGF-1 is also associated with increased myelination [42], which may reduce the progression of white matter damage. Overall, evidence suggests that resistance training may be particularly protective for people with SIVCI.

Therefore, we propose a proof-of-concept, single-blind RCT to primarily examine the efficacy of a 12-month, twice-weekly PRT program to improve cognitive function and reduce WMH progression in community-dwelling adults with SIVCI. The secondary objective is to assess the effect of PRT on regional brain volumes, white matter integrity, myelin content, functional connectivity, specific cognitive processes, physical performance, cardiometabolic risk factors, sleep, physical activity, mood, quality of life, blood biomarkers, and cortisol from saliva. The tertiary objective is to explore the underlying mechanisms by which PRT may promote cognitive function.

## Methods

### Design

**Fig. 1:**
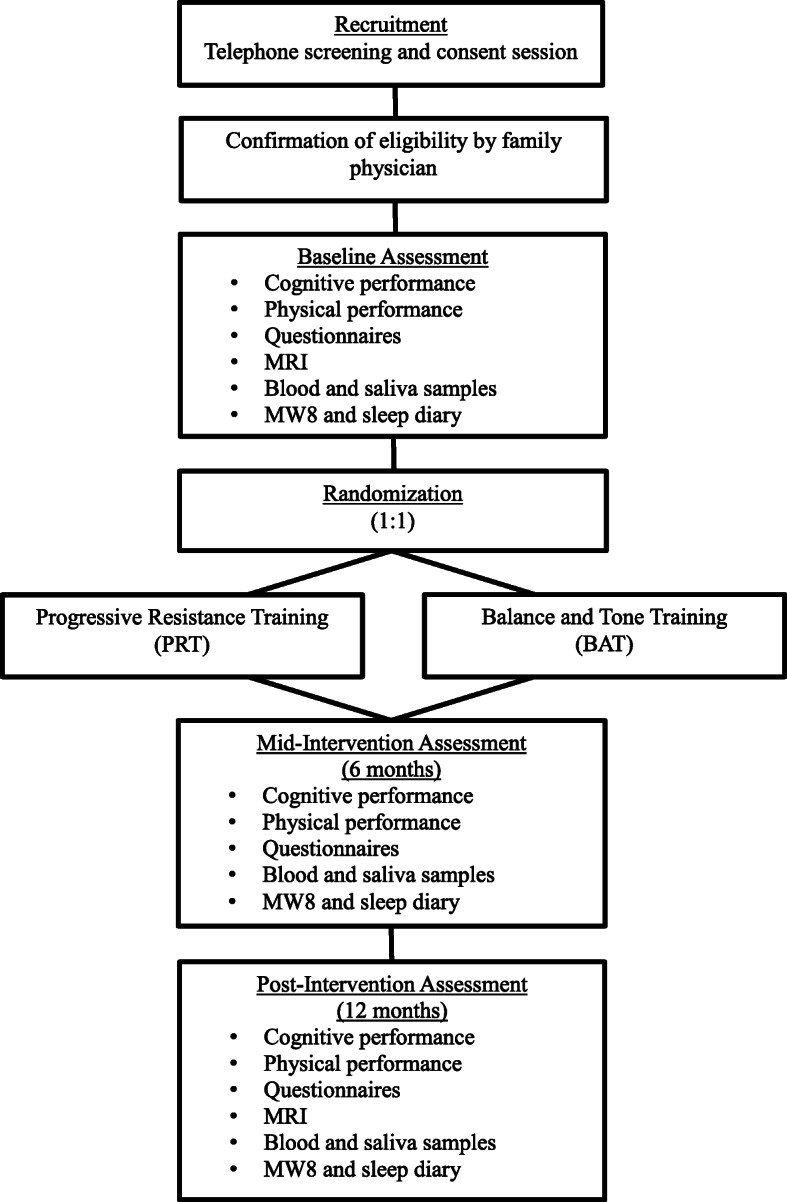
Overview of study design from recruitment to study completion

**Fig. 2:**
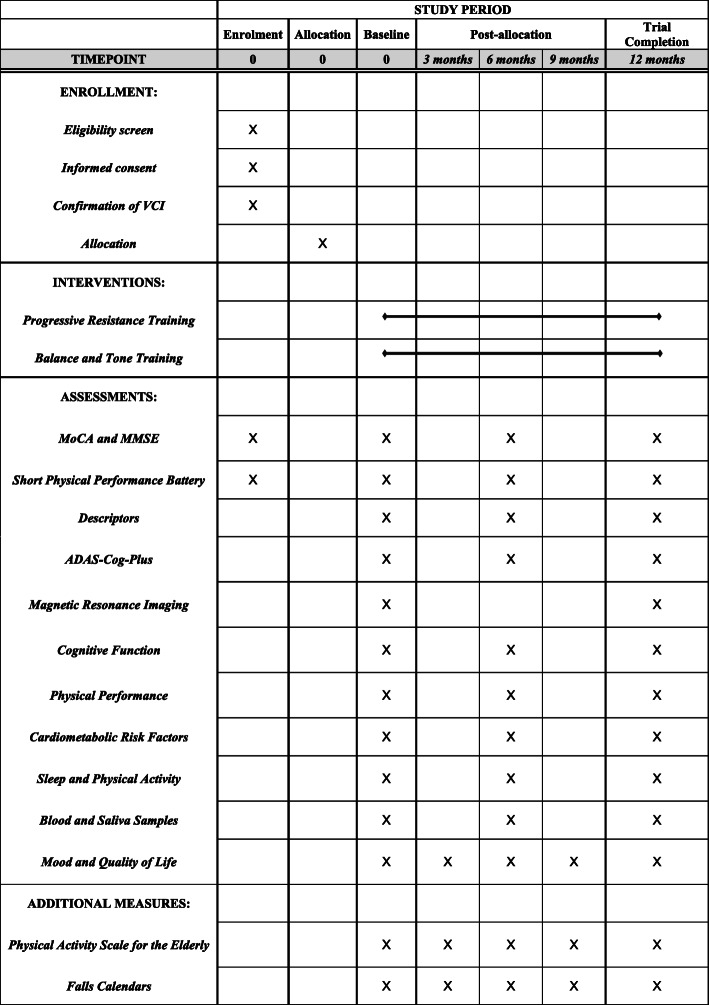
Schedule of enrolment, interventions, and assessments according to the SPIRIT Checklist

We will conduct a 12-month, parallel group, proof-of-concept RCT of 88 community-dwelling adults with SIVCI, aged 55 years and older. Participants will be randomly assigned to receive 12 months of (1) twice-weekly PRT or (2) twice-weekly balance and tone training (BAT; active control). There will be three measurement points occurring at baseline, 6 months, and 12 months conducted by blinded assessors (Fig. 1). The experienced research team will implement standardized protocols and train study personnel (Fig. 2).

### Recruitment

We will recruit adults with SIVCI through general newspaper advertisements and four clinics: (1) University of British Columbia (UBC) Hospital Clinic for Alzheimer Disease and Related Disorders, (2) Vancouver General Hospital (VGH) Stroke Clinic, (3) VGH Falls Prevention Clinic, and (4) VGH Geriatric Internal Medicine Teaching Clinic. Individuals that appear eligible will be mailed an information package regarding the study, including a consent form. Those that are interested will be invited to a screening and consent session. This session will involve the administration of (1) the Physical Activity Readiness Questionnaire [44] to assess physical readiness for exercise and (2) the Mini-Mental State Examination (MMSE) [45] and Montreal Cognitive Assessment (MoCA) [46] to assess cognitive function. When a participant reports a relevant health concern or condition (e.g., arterial fibrillation), they will be provided a form to be completed by their family physician to confirm their current health status and suitability to start an exercise program (Fig. 1).

### Time frame

Participant enrollment began on May 17, 2016, and the final assessment is anticipated to be completed by March 2022. The COVID-19 pandemic impeded recruitment for much of 2020. As of October 24, 2020, 69 individuals (78% of the target sample) have been recruited and randomized.

### Eligibility

#### Inclusion criteria

We will include community-dwelling adults who fulfill the criteria for SIVCI, defined as the presence of cognitive impairment [46] combined with cerebral small vessel disease [47]. Cognitive impairment will be operationalized as a MoCA score < 26 [46], and cerebral small vessel disease will be defined as the presence of WMHs and/or lacunes on computed tomography (CT) or magnetic resonance imaging (MRI) [47]. Additional inclusion criteria require the participant to: (1) be 55 years or older; (2) have an MMSE score > 20 [45]; (3) be community-dwelling (i.e., living in their own homes) in metro Vancouver; (4) be able to comply with scheduled visits, treatment plan, and other trial procedures; (5) read, write, and speak English with acceptable visual and auditory acuity; (6) be on a fixed dose of cognitive medications that is not expected to change during the 12-month study period, or, if they are not on any of these medications, they are not expected to start them during the 12-month study period; (7) provide informed consent; (8) be able to walk independently; and (9) must be in sufficient health to participate in the PRT program.

#### Exclusion criteria

We will exclude individuals who: (1) have an absence of cerebral small vessel disease on a brain CT or MRI; (2) are diagnosed with dementia of any type or another type of neurodegenerative or neurological condition (e.g., Parkinson’s disease); (3) are diagnosed with a genetic cause of SIVCI; (4) are at high risk for cardiac complications during exercise and/or unable to self-regulate or to understand the recommended activity level; (5) participated in regular resistance training in the last 6 months; (6) have clinically important peripheral neuropathy or severe musculoskeletal or joint disease that impairs mobility; (7) recently started taking medications (< 3 months prior to study) that may negatively affect cognitive function, such as anticholinergics; (8) are planning to participate, or already enrolled in, a clinical drug trial or exercise trial concurrent to this study; or (9) are unable to meet MRI scanning requirements, as specified by the UBC 3T MRI Research Center.

### Sample size calculation

Power analyses were conducted in G*Power 3.1 [48]. We have sized this trial to allow the evaluation of PRT on the Alzheimer's Disease Assessment Scale-Cognitive-Plus (ADAS-Cog-Plus) [49] and WMH progression at trial completion; WMH progression is considered a valid surrogate marker in therapeutic trials of SIVCI [50]. The ADAS-Cog-Plus is the original ADAS-Cog-13 with additional measures of executive functions and verbal fluency, and it is more sensitive to subtle cognitive changes than the original ADAS-Cog-13 or ADAS-Cog-11 [49]. Our prior 6-month RCT of aerobic exercise on cognitive function, as measured by the ADAS-Cog-11, in adults with SIVCI observed an effect size of 0.89 (Cohen’s *d*) in the complete-case analysis [51]. We also demonstrated twice-weekly PRT slowed WMH progression in older women; the effect size observed was 0.60 (Cohen’s *d*). Based on this effect size of 0.60, assuming an alpha of 0.05 (two-tailed) and a beta of 0.20, 35 participants per group will provide a power of 0.80 [48]. We are aiming to recruit a total of 88 participants with SIVCI (i.e., 44 participants per group), which will accommodate a conservative 20% drop-out rate.

### Data entry

No personal identifiers will be acquired during data collection. All paper-based data will be stored in locked cabinets and all alphanumeric data will be entered by a trained study personnel who will conduct range checks for data values. All alphanumeric data will be stored on a secured server hosted by the University of British Columbia. All data will be deidentified.

### Measurements

#### Descriptors

Body mass will be measured in kilograms using a calibrated digital scale and height in centimeters using a wall-mounted stadiometer. General health, medication use, and socioeconomic status will be ascertained using questionnaires. The Functional Comorbidity Index [52] will be used to estimate the degree of comorbidity associated with physical functioning.

#### Primary outcomes

The study will be powered based on expected changes in cognition and WMH progression. Improvement in either outcome is considered evidence of efficacy.

##### Alzheimer’s Disease Assessment Scale-Cognitive-Plus

Change in global cognitive function will be measured by the ADAS-Cog-Plus using a multidimensional item response theory model, which utilizes item scores from multiple cognitive assessment instruments to generate a global cognitive function score and standard error of measurement for that score. The ADAS-Cog-Plus includes the original 13-item assessment of memory, language, and praxis [53] with additional measures of executive functions and verbal fluency. Executive functions are assessed using the: (1) Trail Making Test (parts A and B) [54], a measure of set-shifting; (2) verbal digit span forward and backward [55], a measure of working memory; (3) Digit Symbol Substitution Test [56], a measure of working memory and psychomotor speed; and (4) category fluency [57], a measure of semantic and working memory. Higher scores on the ADAS-Cog-Plus indicate poorer cognitive performance.

##### White matter hyperintensity quantification

Brain MRI scans will be acquired at baseline and trial completion (12 months) at the UBC MRI Research Center. Neuroimaging will be performed on a Philips 3.0-Tesla Achieva scanner (Best, The Netherlands) with an 8-channel phased array head coil. Three-dimensional (3D) T_2_-weighted (T_2_-w) and proton density-weighted (PD-w) structural MRI scans will be acquired to quantify WMH volume.

For detailed information on MRI sequence parameters, please refer to Table 1.

**Table 1:** Magnetic resonance imaging protocol

	3D T_1_-w MPRAGE^a^	3D T_2_-w	PD-w	DWI^b^	GRASE	rs-fMRI
Resolution acquired/reconstructed (mm^2^)	1 × 1/1 × 1	1 × 1/0.8 × 0.8	0.99 × 1/0.98 × 0.98	2.24 × 2.24/2 × 2	0.99 × 2.04/0.96 × 0.95	3 × 3/3 × 3
Slice thickness acquired/reconstructed (mm)	1/1	1.6/0.8	1/1	2.20/2.20	5/2.5	3/3
Number of reconstructed slices	170	200	170	70	40	36
Field of view ap/rl/fh (mm)	256 × 200 × 170	256 × 160 × 256	250 × 170 × 250	224 × 224 × 154	230 × 190 × 100	240 × 240 × 143
Orientation	Transverse	Sagittal	Sagittal	Transverse	Transverse	Transverse
Echo time (ms)	3.5	363	30	60	8, 16, 24 … 384	30
Repetition time (ms)	1800	2500	3000	7.1	1073	2000
Flip angle (deg)	8	90	90	90	90	90
Acquisition time (min)	6:34	4:43	10:33	7:27	7:30	5:00
Purpose	Anatomical reference	WMH quantification	WMH quantification	White matter integrity quantification	Myelin quantification	Functional connectivity assessment

*ap* anterior-posterior, *rl* right left, *fh* foot-head

^a^Additional sequence parameters for 3D T_1_-w MPRAGE: inversion time = 810 ms

^b^Additional sequence parameters for DWI: 60 gradient directions at *b* = 700 s/mm^2^, 1 unweighted scan

T_2_-w and PD-w images will be preprocessed using standard and publicly available neuroimaging tools that include: (1) MR intensity inhomogeneity correction using a multiscale version of the nonparametric non-uniform intensity normalization method (N3) [58]; (2) a structure-preserving noise-removal filter (SUSAN) [59]; and (3) all non-brain tissues will be removed using the brain extraction tool (BET) [60].

WMHs will then be identified and digitally marked by a radiologist/neurologist with experience in WMH identification. The radiologist/neurologist will be blinded to all participant information, including treatment assignment. Baseline and 12-month scans will be co-registered and reviewed together to ensure consistency of identification of small lesions across time. The radiologist/neurologist will use the following guidelines in the seeding procedure, which was designed to be efficient and intuitive: (1) mark all distinct WMH regardless of size; (2) place more than one point on a lesion if the additional points would help define the extent of the lesion; and (3) place at least one point near the center of each lesion [61].

The seeded images will then be segmented by a method that automatically computes the extent of each marked lesion to create a lesion mask [61]. This segmentation method has been validated in large data sets with a wide range of lesion loads. It was found to be highly accurate compared to manual radiologist segmentations and also robust to variations in the placement of seed points [61]. Full details on the point placement procedure and subsequent automatic segmentation are described in previous work [61]. The lesion masks were then used to quantify WMH volume in cubic millimeters (mm^3^). All lesion masks will be reviewed by a trained research assistant to ensure accuracy.

#### Secondary outcomes

##### Brain magnetic resonance imaging

In addition to WMH volume, measures of brain structure and function will be acquired. Following a survey and reference scan, the following sequences will be collected: (1) 3D T_1_-weighted (T_1_-w) with an inversion recovery magnetization-prepared rapid acquisition with gradient echo (MPRAGE) sequence for anatomical reference; (2) diffusion-weighted imaging (DWI) acquired with high angular resolution to quantify white matter integrity; and (3) whole-brain 48-echo gradient and spin echo (GRASE) for T_2_ measurement to quantify myelin [62].

In addition, a subset of participants will also undergo an 8-min resting-state functional MRI (rs-fMRI) to assess changes in the connectivity of large-scale functional networks. During this scan, participants will be asked to rest with their eyes open while looking at a fixed point and to think of nothing in particular. The rs-fMRI scan will be used to establish the relevance of lesion location on changes in functional connectivity, in conjunction with a technique called lesion network mapping [63, 64]. Within this technique, the location of each WMH will be overlaid onto the functional connectivity of a human connectome. This will identify the level of overlap between the functional connectivity associated with the location of the WMHs and that of the large-scale functional networks. For detailed information on MRI sequence parameters, please refer to Table 1.

##### Cognitive function

The ADAS-Cog-13 is a 13-item assessment of memory, language, and praxis [53]. Scores range from 0 to 85, with higher scores indicating greater cognitive impairment. Three key executive processes will be assessed: (1) set-shifting; (2) working memory; and (3) selective attention and conflict resolution. We will measure set-shifting using the Trail Making Test (parts A and B) [54]. Working memory will be assessed by the verbal digit span forward and backward tests [57]. The Digit Symbol Substitution Test will measure psychomotor speed and working memory. The Stroop Color-Word Test [65] will measure selective attention and conflict resolution. The Picture Sequence Memory Test from the National Institute of Health Toolbox [66] will measure episodic memory. Participants will see a sequence of pictured activities presented in a specific order and are asked to put the pictures back into the order that was demonstrated. At practice, participants will be presented with four pictures, and for the main task, they will be presented with a sequence of 12 and then 16 pictures.

##### Physical performance

**Balance and mobility**

The Short Physical Performance Battery [67] includes standing balance (i.e., side-by-side stand, semi-tandem stand, and tandem stand), 4-m walk test, and repeated chair stands. Each component is rated from 0 (inability to perform the task) to 4 (optimal performance), for a maximum of 12 points. A score < 9 is predictive of subsequent disability [67].

**Functional mobility**

The Timed-Up-and-Go (TUG) test is a timed assessment that requires participants to stand up from a chair, walk 3 m at their usual speed, turn, walk back to the chair, and sit back down [68]. We will assess performance on the standard TUG test and on a dual-TUG test, whereby participants will be asked to subtract sevens from a randomly given number, while performing the task [68, 69]. A subset of participants will also complete a dual-task walking assessment. Using a GAITRite mat [70], participants will be asked to: (1) name as many items from a given category (category fluency) in 30 s while standing; (2) walk at their usual pace along the mat; and (3) walk at their usual pace while completing the category fluency task. Dual-task cost is calculated as (dual-task time − walking time)/walking time. A lower score is indicative of better dual-task performance.

**Physiological fall risk**

We will use the Physiological Profile Assessment© (PPA) [71] to assess physiological fall risk. The PPA has a 75% predictive accuracy for falls in older adults [71]. It is composed of five physiological domains: (1) postural sway, (2) hand reaction time, (3) dominant quadriceps strength, (4) proprioception, and (5) edge contrast sensitivity. A PPA *z*-score of 0–1 is indicative of mild fall risk, 1–2 indicates moderate risk, 2–3 indicates high risk, and > 3 indicates marked risk.

**Functional capacity**

We will assess functional capacity using the six-minute walk test [72]. Participants will be asked to walk as far as they can in 6 min, breaks included. We will record blood pressure immediately before and after the walk, and participants will be asked to rate their walk on the Borg Rating of Perceived Exertion scale [73]. Performance will be recorded as the number of meters walked in 6 min.

**Muscular strength**

We will assess upper body strength using maximal hand grip strength measured by a dynamometer. For the lower body, we will measure dominant quadriceps (isometric) strength using a simple strain gauge to the nearest 0.5 kg. In a subset of participants, lower body strength will be assessed using a Biodex System 4 Pro™ dynamometer. Maximal torque at a velocity of 60°/s and 180°/s will be recorded for both knee flexion and extension.

##### Cardiometabolic risk factors

We will assess: (1) resting systolic and diastolic blood pressure; (2) blood biomarkers (e.g., serum glucose and lipid profile), collected from fasted blood samples; (3) body mass index (BMI), using the formula mass (kg)/(height (m)^2^); (4) waist-to-hip ratio, by measuring hip and waist circumference in centimeters; and (5) arterial stiffness as measured by carotid-femoral pulse-wave velocity using the Complior system (Alam Medical, France).

##### Sleep and physical activity

Subjective sleep quality will be measured by the Pittsburgh Sleep Quality Index, a self-rated questionnaire assessing sleep disturbances over a 1-month period [74]. Objective measures of sleep quality will be estimated over a 2-week period using the MotionWatch8© (MW8) wrist-worn actigraphy unit (CamNtech; Cambridge, UK) to estimate sleep duration, latency, and fragmentation. Participants will also be asked to complete the Consensus Sleep Diary each morning [75]. In addition to sleep quality, the MW8 will also be used to calculate daily physical activity. The number of minutes spent in moderate to vigorous physical activity (> 3.0 METs) is compared to the total time spent awake and out of bed to determine the percentage of each day spent in physical activity [76].

##### Mood and quality of life

Mood will be measured using the Centre for Epidemiologic Studies Depression Scale [77], which is a 20-part questionnaire that asks how often over the past week symptoms associated with depression were experienced. It is scored out of 60 with higher scores indicative of greater depressive symptoms. The ICEpop CAPability Measure for Older adults [78, 79] will be used to assess wellbeing across five attributes: (1) attachment (love and friendship); (2) security (thinking about the future without concern); (3) role (doing things that make you feel valued); (4) enjoyment (enjoyment and pleasure); and (5) control (independence).

##### Economic evaluation measures

The EuroQol-5 Dimension-5 Level (EQ-5D-5L) questionnaire [80] compiles a composite score (i.e., health state utility value) from ratings of perceived health across five domains: (1) mobility; (2) self-care; (3) usual activities; (4) pain/discomfort; and (5) anxiety/depression. A health resource utilization questionnaire will quantify health care system-related costs during the study period [81].

##### Blood and saliva samples

In a subset of participants who consent, fasting blood samples will be collected in the morning by standard venipuncture at baseline, 6, and 12 months. Blood will be processed and stored at – 80 °C as plasma, serum, and whole blood. The main analytes of interest include pro and mature BDNF, IGF-1, VEGF, Cathepsin-B, irisin, adiponectin, sex steroid hormones (estradiol, estrone, testosterone), s100-B, and pro- and anti-inflammatory cytokines (e.g., IFN-γ, IL-1β, IL-4, IL-6, IL-10, IL-12p70, IL-13, IL-17, TNF-α, Rantes, CXCL1, IL-18, TGF-β). To examine potential genetic moderation of exercise efficacy, we will examine the BDNF Val66Met polymorphism, a common single-nucleotide polymorphism within the pro-domain region of the human BDNF gene resulting in an amino acid substitution of valine (Val) to methionine (Met) at position 66. We will also obtain the apolipoprotein E genotype. DNA will be extracted from whole blood by standard protocol and genotype will be determined by a TaqMan by-design assay. Remaining blood samples will be stored for future analyses.

In a subset of participants who consent, saliva samples will be taken to assess hypothalamic–pituitary–adrenal axis activity. Specifically, we will examine: (1) total cortisol concentration over the day (area under the curve); (2) cortisol awakening response, a distinct aspect of the circadian cortisol profile; and (3) changes in cortisol response to engaging in an exercise class with time of day held constant. Free cortisol levels will be assessed in salivary samples (Salivette®) collected 5× each day (at awakening, 30 min after awakening, 2 pm, 4 pm, and bedtime) for 2 consecutive days at each measurement point. Also, salivary samples will be collected immediately before and immediately after the first exercise class and the last exercise class to examine cortisol response to exercise.

#### Treatment allocation

##### Randomization

Participants will be randomly assigned (1:1) to either the 12-month, twice-weekly PRT program or the 12-month, twice-weekly BAT program. The randomization sequence will be generated and held by a central web-based randomization service. Permuted blocks of varying sizes will be used to ensure balance over time.

##### Allocation concealment

Participant recruitment and enrollment will be managed by research personnel who will screen for eligibility, acquire consent, and conduct baseline assessments. Randomization to an intervention group will occur after completion of the baseline assessments. Research personnel conducting assessments and data analysis will be blinded to group allocation so unblinding will not occur. We will not be able to blind participants or personnel delivering the interventions and obtaining the monthly physical activity data (see intervention compliance). Blinding will be supported by providing explicit instructions to the research personnel and participants not to discuss issues related to physical activity during the assessments.

#### Experimental groups

All classes will be led by certified instructors with a participant to instructor ratio of 4:1. Each 60-min class will include a 10-min warm up (i.e., stretching of the major muscles and walking on the spot), 40-min of training, and a 10-min cool down (i.e., stretching and relaxation techniques). Every instructor will be audited on a monthly basis by the exercise class coordinator to ensure consistent protocol delivery.

##### Progressive resistance training

The PRT program will consist of a combination of free weight exercises including mini-squats, mini-lunges, and lunge walks and pressurized air system exercises including biceps curls, seated row, latissimus dorsi pull downs, triceps extension, leg press, hamstring curls, and calf raises. The intensity of training will begin at 50–60% of their 1 repetition maximum (1RM) and progress to 2 sets of 6–8 repetitions at 75–85% 1RM by week 4. The 7RM method will be used to increase the training load when 2 sets of 6–8 repetitions are completed with the correct form. The number of sets completed and the load lifted will be recorded for each participant at each class.

To meet the public health mandates of COVID-19, when it is necessary, training will occur at home, with the use of resistance bands of various weights. Participants will be provided access to instructional videos by either YouTube or DVD. They will be called on a weekly basis to monitor progress and compliance.

##### Balance and tone training

The BAT program will consist of stretching, basic core/kegal exercises, deep breathing, and relaxation techniques. Other than body weight, no additional loading will be applied to any of the exercises. This training has not been shown to improve cognitive function [82] and thus will serve as a control for confounding variables such as physical training received by traveling to the training centers, social interaction, and changes in lifestyle secondary to study participation.

To meet the public health mandates of COVID-19, when it is necessary, training will occur at home, with the use of small equipment (e.g., Pilate ball). Participants will be provided access to instructional videos by either YouTube or DVD. They will be called on a weekly basis to monitor progress and compliance.

#### Intervention compliance

Class attendance will be recorded by the instructors and compliance will be defined as the percentage of the total classes attended. To promote adherence to the exercise program, we will implement strategies including: (1) regular contact; (2) developing coping and action plans; (3) setting implementation intentions and concrete plans; and (4) encouraging participants to self-monitor their progress. Monthly monitoring of extracurricular physical activities will also be performed by an unblinded assessor using the Physical Activities for the Elderly [83, 84] questionnaire throughout the study. In addition, at each measurement point, physical activity over 14 days will be quantified using data acquired by the MW8 [76].

#### Data and adverse event monitoring

A Data and Safety Monitoring Committee will be established by co-investigators who will be independent from the day-to-day conduct of the study. They will review all adverse events on a monthly basis and stop the study if the data are of sufficient concern (e.g., increased rate of falls as a result of the intervention). All adverse events will be reported to this committee and to the relevant university and health authority ethics boards by the study coordinator. There is no anticipated harm and compensation for trial participation.

### Statistical analyses

The analyses will follow the intention-to-treat principle (i.e., all individuals will be analyzed according to their group allocation regardless of compliance). We will evaluate between-group differences (PRT vs. BAT) in ADAS-Cog-Plus and WMH volume, adjusted for baseline score, using mixed linear models. Restricted maximum likelihood estimation will be used in order to include all randomized participants to estimate treatment effects, regardless of loss to follow-up. Time since baseline (6 months versus 12 months) will be considered as a repeated variable and will be included as a fixed effect in addition to group and group-by-time interaction. Baseline score on the outcome variable and participant baseline characteristics will be included as fixed covariates. The intercept will be specified as a random effect. Primary and secondary outcomes will be analyzed using this same analytic model.

The intention-to-treat analysis will be followed by a complete-case analysis, in which only participants with valid data at all time points will be included. Moreover, we will compare participants in the PRT group who are compliant with the intervention (defined as attending at least 60% of the total exercise sessions) with the BAT group.

To explore underlying mechanisms, we will assess the association between changes in cognitive function with changes in: (1) neuroimaging outcomes; (2) cardiometabolic risk factors; (3) blood biomarkers; and (4) saliva biomarkers.

The economic evaluation will examine the efficiency of the 12-month PRT program compared with BAT. The outcome of our cost–utility analysis is the incremental cost–utility ratio (ICUR): ICUR = ∆Cost/∆ quality-adjusted life years (QALYs); QALYs, estimated from the EQ-5D-5L, represent time spent in given health states [85, 86].

## Discussion

Although previous research has highlighted the benefit of aerobic training for people with SIVCI [25], further research is required to determine the effect of resistance training in this population. Consequently, the findings from this RCT will provide important insight into the efficacy of resistance training on improving cognitive function and slowing WMH progression in adults with SIVCI. In addition, our findings will provide greater understanding of the underlying mechanisms by which PRT promotes cognitive function in SIVCI.

### Public health

The 2020 Lancet Commission on dementia prevention, intervention, and care reports a small, beneficial effect of physical activity on normal cognition, with a possible effect in mild cognitive impairment. This conclusion was mostly based on studies of aerobic exercise, and it was noted that evidence about the effect of other types of exercise, specifically PRT, on dementia risk is scarce [87]. Given the dearth of high-quality studies of PRT, the findings from this RCT will provide new insights into the efficacy of PRT on improving cognitive function and slowing WMH progression in adults with SIVCI. In addition, our findings will aid understanding of the underlying biological mechanisms by which PRT may be exerting its effects in SIVCI. Strategies to improve cardiovascular and cerebrovascular health will preserve independent functioning and quality of life in adults with SIVCI. Moreover, establishing the efficacy of different types of exercise training will add substantially to options for exercise prescription for adults with SIVCI. These efforts are critical at this point in time as cognitive impairment and dementia pose an enormous socioeconomic burden, negatively affecting families, communities, and health care systems.

### Trial status

This protocol is version nine, updated February 21, 2021. Participant enrollment began on May 17, 2016, and recruitment is anticipated to be completed by March 2021. Any changes to the protocol will be documented by the principal investigator and all research personnel will be notified. The clinical trial registration will be amended for all updates to the protocol.

## Supplementary Information

**Additional file 1.**

**Additional file 2.** SPIRIT 2013 Checklist.

## Data Availability

Only investigators and research teams with ethical approval will have access to the final datasets. The datasets used and/or analyzed during the current study will be available from the corresponding author on reasonable request.

## Abbreviations

SIVCI: Subcortical ischemic vascular cognitive impairment
WMHs: White matter hyperintensities
RCT: Randomized controlled trial
PRT: Progressive resistance training
BAT: Balance and tone
BDNF: Brain-derived neurotrophic factor
IGF-1: Insulin-like growth factor-1
VEGF: Vascular endothelial-derived growth factor
MMSE: Mini-Mental State Examination
MoCA: Montreal Cognitive Assessment
ADAS-Cog: Alzheimer’s Disease Assessment Scale-cognitive
1RM: One repetition maximum
