# Supplementary material for: Reshaping the path of vascular cognitive impairment with resistance training: a study protocol for a randomized controlled trial
Source: Trials. 2021 Mar 18;22:217. doi: 10.1186/s13063-021-05156-1 (PMC7971404; doi:10.1186/s13063-021-05156-1)
Supplement: Supplementary file 1 — Additional file 1. [file 13063_2021_5156_MOESM1_ESM.doc]

**T H E U N I V E R S I T Y O F B R I T I S H C O L U M B I A**


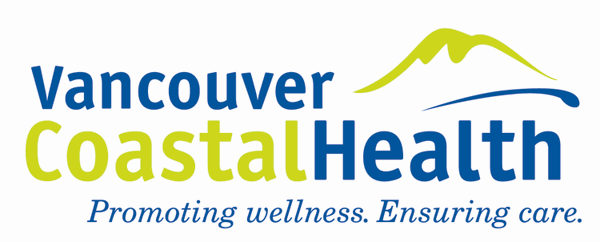

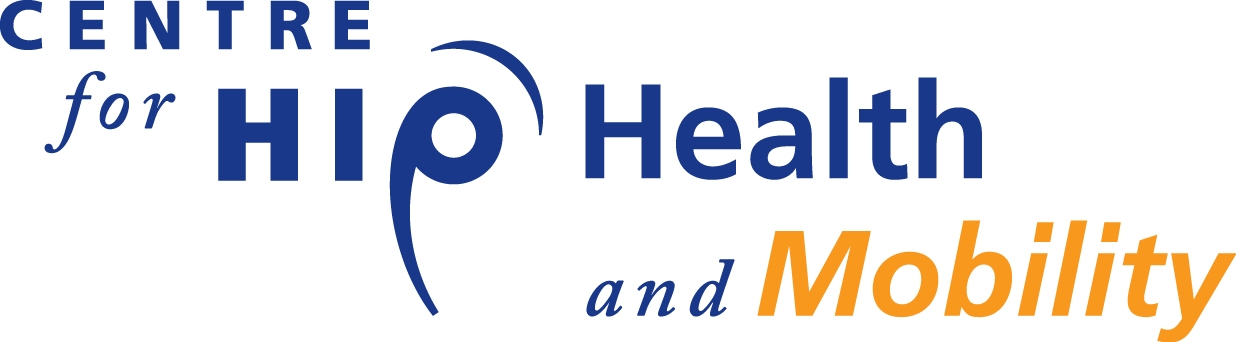

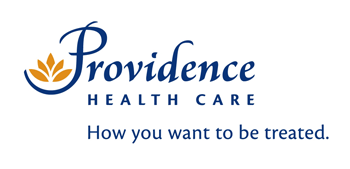


**SUBJECT INFORMATION AND CONSENT FORM**

**Reshaping the Path of Vascular Cognitive Impairment with Resistance Training**

**Principal Investigator:** Teresa Liu-Ambrose

Professor, Department of Physical Therapy

Faculty of Medicine

Centre for Hip Health and Mobility

Phone Number: (604) 875-4111 x 69059

Fax Number: (604) 822-1870

**Co-Investigators:** John Best, Jennifer Davis, Charlie Goldsmith, Ging-Yuek Robin Hsiung, Roger Tam, Thalia Field, Kenneth Madden, Alex MacKay, Cindy Barha

**Study Team members:** Elizabeth Dao, Chun Liang Hsu, Lisanne ten Brinke, Stephanie Doherty, Madison Welch, Joey Chan

**Sponsors:** This research project is funded by the Heart and Stroke Foundation of Canada and Michael Smith Foundation for Health Research.

**Emergency contact number:** In case of an emergency, please contact the UBC Hospital switchboard at (604) 822-7121 and ask for the physician on-call for the University of British Columbia Hospital Clinic for Alzheimer Disease and Related Disorder, 24 hours a day, 7 days a week.

**If you are a substitute decision-maker for someone who may take part in this study, permission from you and the agreement and the assent (agreement) of the potential research participant may be required. When we say “you” or “your” in this consent form, we mean the research participant; “we” means the doctors and other research staff.**

**GENERAL INFORMATION**

You are being invited to participate in this research study because you may be eligible for a research study exploring whether a resistance training exercise program or a balance and toning exercise program will assist in maintaining cognitive function. We are specifically seeking 88 individuals who are demonstrating cognitive changes and have vascular issues (i.e., memory complaints and a history of high blood pressure or cholesterol, diabetes, or transient ischemic attacks). **If you are interested in participating in the research, please be advised that further screening by a physician or study team member will take place to determine your eligibility.**

**Your participation in this study is entirely voluntary, so it is up to you to decide whether or not to take part in this study.** Before you decide, it is important for you to understand what the research involves. This consent form will tell you about the study, why the research is being done, what will happen to you during the study and the possible benefits, risks and discomforts.

This consent form tells you about the research study and what will happen if you decide to take part in it. You may take home an unsigned copy of this consent form to think about or discuss with friends, relatives and/or your family doctor. You need to understand the risks and benefits of the study. **Please take time to read the following information carefully** before you decide whether or not to participate. At any time before or during the study, please ask the principal investigator or study coordinator to explain any words or information that you do not understand.

Once you have read this consent form and your questions have been answered, you will be asked to sign and date the last page if you want to participate in this study. By signing this consent form, you are stating that you agree freely and voluntarily to participate in this study.

If you do decide that you would like to participate, you are still free to withdraw at any time and without giving any reasons for your decision. If you do not wish to participate, you do not have to provide any reason for the decision nor will you lose the benefit of any medical care to which you are entitled or presently receiving.

This study is being conducted by Drs. Teresa Liu-Ambrose (Principal Investigator), John Best, Jennifer Davis, Ging-Yuek Robin Hsiung and Roger Tam from the University of British Columbia, and Charlie Goldsmith from Simon Fraser University. The Heart and Stroke Foundation (HSF) of Canada is funding this study. The Principal Investigator has received financial compensation from HSF for this study (e.g., to pay the costs of equipment, procedures and staff). Dr. Liu-Ambrose does not receive any personal financial compensation for this study. You are entitled to request any details concerning this compensation from the Principal Investigator.

In recent years, scientists have been interested in physical activity as the main reason to slow down the progress cognitive decline. A large number of research studies have been done to shown that regular physical activity can reduce or slow down memory complaints. However, more work is needed to examine the comparative effects of exercise training among individuals who have mild changes in their cognition due to underlying vascular issues (e.g. high blood pressure, diabetes).

**PURPOSE OF THE STUDY**

The main goal of the proposed study is to determine whether a resistance training exercise program or a balance and tone exercise program will combat cognitive decline.

You can participate if you meet the criteria for this study and are at least 55 years of age and not living in a nursing or extended care facility.

You should not participate in this study if you: 1) have been diagnosed with any other neurological condition that affects cognition or mobility such as Parkinson’s or Multiple Sclerosis; 2) are at high risk for cardiac complications during exercise and/or unable to self-regulate activity or to understand recommended activity level; 3) have peripheral neuropathy (i.e., muscle weakness, autonomic changes, and sensory changes) or severe musculoskeletal or joint disease that impairs mobility (i.e., severe arthritis); 4) are taking medications that negatively affect cognitive function, such as anticholinergics, major tranquilizers or anticonvulsants (i.e., amitriptyline, antipsychotics, gabapentin, valproic acid); 5) you plan to participate or are enrolled in a clinical drug trial; 6) have aphasia (i.e., inability to speak) as judged by an inability to communicate by phone; or 7) have ongoing drug or alcohol use or dependence that may affect your ability to comply with study procedures.

You cannot participate in the MRI scanning if you have any of the following:

- Cardiac pacemaker, wires, or defibrillator;
- Metal in eye or orbit;
- Ferromagnetic aneurysm clip;
- Claustrophobia; or
- Pregnancy.

**STUDY PROCEDURES**

If you agree to be in this study, you will have a 45 minute first visit (screening) where a study team member will ask you to answer some questions to see if you are able to participate in this study. If there are questions that you are not comfortable answering, you do not need to answer them. If necessary, you may see physician Dr. Hsiung or Dr. Madden (or their delegates) for further screening. **You may be excluded from this study after this screening session.**

If you are considered eligible to participate in this study after the screening session, you should expect the following:

There will be 3 additional visits – one visit at the beginning of the study, one visit at 6 months of study, and one at 12 months (end of study). Each visit will take about 3 hours and you will have your height and weight measured, complete some questions regarding my general health, use of health care resources, quality of life, mood, and physical activity level. If there are questions that make you feel uncomfortable, you do not have to answer them. You will also be asked to complete some tasks to see how well you can remember, pay attention, and problem solve, and how well you can balance. Your walking ability will be assessed on how far and quickly you can walk and how you are able to move around.

You will be randomly assigned to one of two groups: 1) Resistance Training Exercise Program; or 2) Balance Exercise and Tone Program. Randomly assigned means that you have an equal chance of being in any group – this is like flipping a coin. If you are selected into the Exercise Training group or the Balance and Tone Program, you will be asked to participate in an additional 104 hours of training over a 12 month period of the study. Description of both groups can be found below.

***Exercise Program***

If you are assigned to the exercise group you will be asked to attend 1 hour classes 2 times per week for 52 weeks. You will be completing a range of exercises, such as standing on one leg or lifting a weight with your arm/leg, with other people and group instructors based on your individual level. Your caregiver(s), family, or friends are welcome to join these classes. The instructors will tell you how to perform all exercises, how many times to perform each exercise and monitor your performance. You will also be telling the instructors how you feel during your exercise sessions.

***Balance Exercise and Tone Program***

If you are assigned to the Balance Exercise and Tone group you will be asked to attend 1 hour classes 2 times per week for 52 weeks. You will be completing stretching exercises, learn relaxation techniques, and balance exercises with other people and group instructors based on your individual level. Your caregiver(s), family, or friends are welcome to join these classes. The instructors will tell you how to perform all exercises, how many times to perform each exercise and monitor your performance. You will also be telling the instructors how you feel during your exercise sessions.

***Visits to the Centre for Hip Health and Mobility***

At the beginning, mid-way (6 months/26 weeks), and the end of the study (12 months/52 weeks), you will be asked to come to the Centre for Hip Health and Mobility (located at 828 West 10^th^ Ave) for a 3-hour appointment. During these sessions, we will measure your height and weight, ask you to complete questionnaires regarding your general health, use of health care resources, quality of life, mood, and physical activity level. You are not required to answer any question in which you feel uncomfortable doing so.

We will also ask you to perform a number of cognitive tasks (e.g., how well you can remember a string of numbers) and physiological tasks (e.g., how well you can balance). We will also assess your walking ability (both how far you can walk and how quickly you can walk) and your general ability to move around in daily life (e.g., how quickly you can stand up from a chair).

***Monitoring of Sleep***

Improved sleep may be how exercise improve cognitive function. Therefore, at the beginning, mid-way (6 months/26 weeks), and the end of the study (12 months/52 weeks), you will be asked to wear a watch for two weeks that will monitor how much and how well you sleep. During these times, you will also be asked to document how well you think you slept on daily basis. This will take less than 5 minutes per day.

***Monthly Physical Activity Monitoring***

You will be asked to complete a monthly questionnaire to record your physical activity levels.

***Visits to the UBC High Field Magnetic Resonance Imaging (MRI) Centre***

At the beginning and 12-month points in the study, you will be asked to come to the UBC High Field MRI Centre for a 45 minute appointment. During this 45 minute appointment, we will examine your brain structure. You will also be asked to close your eyes and just rest for a duration of 40 minutes in the scanner.

MRI is a non-invasive method of taking pictures of your brain using a standard MRI scanner. You cannot participate in MRI scanning if you have any of the following:

- Cardiac pacemaker, wires, or defibrillator;
- Metal in eye or orbit;
- Ferromagnetic aneurysm clip;
- Claustrophobia; or
- Pregnancy.

In addition, you may not participate if you have the following:

• Artificial heart valve;

• Ear or eye implant;

• Brain aneurysm clip;

• Implanted drug infusion pump;

• Electric stimulator for nerves or bones;

• Coil, catheter, or filter in any blood vessel;

• Orthopedic hardware (artificial joint, plate, screws, rods);

• Other metallic prostheses;

• Shrapnel, bullets, or other metal fragments; or

• Surgery or tattoos (including tattooed eyeliner) in the last six weeks.

If you have any of the above, your individual case will be reviewed by the UBC Hospital MR Technologist and/or Radiologist, and a decision will be made regarding your participation in the study. In many cases, an operative report may be required to assess the nature of the implants in your body.

For the MRI scan, you will lie flat on an automated bed that is moved into a cylinder 1 meter wide scanner. A mirror will be placed above the head to allow you to see out to the scanner. Once inside the scanner, the procedure will take no more than one hour. There will be no discomfort other than having to lie still and hear the noise from the scanner for the duration of the procedure. Headphones and ear plugs will be worn for ear protection. Tingling sensations sometimes occur in the arms, but these can usually be prevented by keeping the hands apart. We will maintain visual and verbal contact with you throughout the MRI scanning sessions. As an extra precaution, you will be given an “emergency” button to be used if you wish to end the procedure. Please note that none of the MRI images taken during the experiment will be reviewed by a qualified radiologist; thus, we can provide no clinical evaluation of any anatomic abnormalities that may exist, nor can we offer any clinical referrals.

As this will NOT be a medically indicated examination, there will be no formal review of the scans and no report. The MRI scan being done is designed to answer research questions, not examine your brain medically. This MRI scan is not a substitute for one a doctor would order. It may not show problems that would be picked up by a medical MRI scan. However, if we believe that we have found a medical problem in your MRI scan, we will ask a doctor who is trained in the reading of MRI scans, a radiologist, to help us review the scan. If the radiologist thinks that there may be an abnormality in your MRI scan, we will contact you and with your permission, contact your family physician and help him or her get the right follow-up for you. No information generated in this study will become part of your record routinely. However, if the study detects an abnormality in your MRI scan and further follow-up is required, then this information may become part of your record.

***Saliva Sampling for Stress Assessment***

We are seeking to determine whether a 12-month group-based exercise training program will combat cognitive decline through alterations in the stress hormone, cortisol. Having too much or too little cortisol can be related to impairments in cognition. Longer-term exercise interventions have been shown to reduce cortisol is some people but not in others and thus, it may be that exercise exerts some of its beneficial effects via reductions in cortisol.

The stress hormone, cortisol, can be found and measured in several tissues, including saliva. Thus you will be asked to provide us with 5 saliva samples at specific times of the day for 2 consecutive days (10 samples). This will occur at 3 timepoints: immediately before you start the exercise classes (beginning of the study), at the midpoint of the study (6 months), and immediately after you end the classes (12 months after beginning the study). In addition, at these same timepoints, we will collect 2 saliva samples in class: 1 immediately before beginning the day’s workout, and the other immediately after the class.

To make this easier, more convenient and more hygienic for you, we will have you collect your own saliva using a research device called a Salivette. We will provide you with the appropriate number of Salivettes and instructions on how to use them. Each Salivette consists of a small synthetic swab, like dentists use, which you will chew on for about a minute until you feel that the swab is soaked with your saliva. You will then return the swab to its container and place it in your freezer until you can return all the samples to us.

Providing saliva samples with the Salivette involves very little risk. Saliva sampling with this device has been shown to be safe, reliable, and non-painful. It is vitally important that you not eat or drink any food or medicine 30 minutes prior to providing a saliva sample. Therefore, you may find this aspect of participation a slight nuisance. Although the swab is sterile and has not been treated with anything, some participants may find the taste of the roll unpalatable.

Your saliva samples will be kept at the Brain Research Centre, 2211 Wesbrook Mall, Vancouver, BC, V6T 2B5 and kept there until your sample is analyzed. Dr. Hsiung will be in charge of these samples. Once analyzed, the samples will be destroyed.

Please indicate below whether you agree or not to partaking in saliva sampling during this study.

I AGREE to provide saliva samples for the purpose of this study.

____________ (Initial)

I DO NOT AGREE to provide saliva samples for the purpose of this study.

____________ (Initial)

***Optional Blood Draw for Biomarkers***

As part of this study, you will be asked if you would like to participate in an optional blood draw. You will be provided with a separate consent form for this optional portion. If you consent, you will be asked to complete a blood draw at the beginning, midpoint (6-month), and end (12-month) of the study. The blood draw itself takes approximately 5 minutes.

If you agree to participate in this study the following amount of your time is required:

1. Screening session at the University of British Columbia; 1 session = 45 minutes
2. Assessment sessions at the Vancouver Coastal Health Research Institute: 3 sessions; 3 hours each = 9 hours
3. MRI sessions at the University of British Columbia; 2 sessions; 45 minutes each = 1.5 hours
4. Exercise Program: 52 weeks x 2 sessions/week x 1 hour/session = 104 hours

OR

Balance Exercise and Tone Program: 52 weeks x 2 sessions/week x 1 hour/session = 104 hours

**POTENTIAL RISKS OF STUDY PARTICIPATION**

**Exercise Program:** Mild soft tissue discomfort (i.e., muscle soreness) may be experienced after you being the exercise program. We will attempt to minimize this risk by ensuring you receive proper warm-up and cool-down sessions and all exercise sessions will be led by certified instructors (i.e., registered with BC Recreation and Parks Association as 3rd Age Fitness Instructors) and that the program is tailored to your individual needs as much as possible.

**Balance Exercise and Tone Program:** Mild soft tissue discomfort (i.e., muscle soreness) may be experienced after you begin the Balance and Tone program. We will attempt to minimize this risk by ensuring you receive proper warm-up and cool-down sessions and all exercise sessions will be led by certified instructors (i.e., registered with BC Recreation and Parks Association as 3rd Age Fitness Instructors) and that the program is tailored to your individual needs as much as possible. Furthermore, there is the potential to lose your balance or become unsteady while participating in balance exercises. We will minimize this risk by ensuring research staff spotters that will prevent you from falling, and performing balance exercises next to a ballet bar or chair that you will be able to hold onto at all times.

In the event of a medical emergency, instructors will contact 911 immediately. All instructors will be certified in emergency first aid, including emergency CPR. For all classes, you will be within a maximum 5-minute drive from the VGH Emergency Department or from UBC Hospital Urgent Care.

In the event you experience a serious side effect during this study during normal business hours, you should immediately contact Dr. Liu-Ambrose at (604) 875-4111 x 69059. If it is after 5:00 pm, a holiday or weekend, you should call the Emergency Contact Number on page 1 of the consent form. In case of a serious medical event resulting from this study, please report to an emergency room and inform them that you are participating in a research study and Teresa Liu-Ambrose (Principal Investigator) can be contacted for further information at (604) 875-4111 x 69059 or 604 617-8047 (cell).

**POTENTIAL BENEFITS OF STUDY PARTICIPATION**

Evidence is mounting from randomized controlled trials that exercise has benefits for cognitive function among seniors. Furthermore, regular exercise is beneficial for overall physical health. Health benefits from socialization may also be experienced from the group programs. You may not directly benefit from participating in this study.

Once your participation in the study is concluded we will provide you with the results from the study. If we find the exercise program does have significant benefits, we will provide all the exercise protocol in lay terms to all study subjects.

**COST/REIMBURSEMENT**

All research-related medical care and treatment and any related tests that you will receive during your participation in this study will be provided at no cost to you. In addition, you will be reimbursed up to $210 of transportation costs to cover your public transit and/or parking expenses (no receipt required). However, you will not be paid for participating in this study.

**Confidentiality**

Your confidentiality will be respected. However, research records and health or other source records identifying you may be inspected in the presence of the Investigator or his or her designate by representatives of the UBC Clinical Research Ethics Board for the purpose of monitoring the research. No information or records that disclose your identity will be published without your consent, nor will any information or records that disclose your identity be removed or released without your consent unless required by law.

You will be assigned a unique study number as a participant in this study. This number will not include any personal information that could identify you (e.g., it will not include your Personal Health Number, SIN, or your initials, etc.). Only this number will be used on any research-related information collected about you during the course of this study, so that your identity will be kept confidential. Information that contains your identity will remain only with the Principal Investigator and/or designate. The list that matches your name to the unique study number that is used on your research-related information will not be removed or released without your consent unless required by law.

Your rights to privacy are legally protected by federal and provincial laws that require safeguards to insure that your privacy is respected. You also have the legal right of access to the information about you that has been provided to the sponsor and, if need be, an opportunity to correct any errors in this information. Further details about these laws are available on request to your study doctor.

To do this research, we need to collect health information that identifies you. We will collect information from activities described in the Procedures section of this form. If the results of this study are published or presented in public, information that identifies you will be removed. If you decide not to sign the form, you cannot be in the study.

Your study-related health information such as which group you have been randomized into will be used at UBC only by Dr. Liu-Ambrose, and members of her research team who are listed on this consent form. This is important to allow members of the research team to communicate about which group you are participating in for this research. Your permission to use and disclose your health information remains in effect until the study is complete and the results are analyzed. After that time, information that personally identifies you will be removed from the study records.

At the end of the 5 year retention period, all paper records will be disposed of in confidential waste or shredded, and all electronic records will be deleted.

**VOLUNTARY NATURE OF THE STUDY**

Your participation in this study is voluntary. You may withdraw from this study at any time without giving reasons. If you choose to enter the study and then decide to withdraw at a later time, you have the right to request the withdrawal of your information collected during the study. This request will be respected to the extent possible. Please note however that there may be exceptions where the data will not be able to be withdrawn for example where the data is no longer identifiable (meaning it cannot be linked in any way back to your identity) or where the data has been merged with other data. If you would like to request the withdrawal of your data, please let your study doctor know. If your participation in this study includes enrolling in any optional studies, or long term follow-up, you will be asked whether you wish to withdraw from these as well.

**CONTACTS AND Questions**

You have read the information in this form. Dr. Liu-Ambrose or her associates have answered your question(s) to your satisfaction. You know if you have any more questions after signing this you may contact Dr. Liu-Ambrose at (604) 875-4111 x 69059, or study coordinator Stephanie Doherty at (604) 875-4111 x69313.

If you have any questions about your rights as a research subject, you may call the Research Participant Complaint Line in the University of British Columbia Office of Research Services by e-mail at [RSIL@ors.ubc.ca](mailto:RSIL@ors.ubc.ca) or by phone at (604) 822-8598. Please reference study number H15-00972 when calling so the Complaint Line staff can better assist you.

Consent

**Title: Reshaping the Path of Vascular Cognitive Impairment with Resistance Training**

My signature on this consent form means:

- I have read and understood the information in this consent form.
- I have had enough time to think about the information provided.
- I have been able to ask for advice if needed.
- I have been able to ask questions and have had satisfactory responses to my questions.
- I understand that all of the information collected will be kept confidential and that the results will only be used for scientific purposes.
- I understand that my participation in this study is voluntary.
- I understand that I am completely free at any time to refuse to participate or to withdraw from this study at any time, and that this will not change the quality of care that I receive.
- I authorize access to my health records as described in this consent form.
- I understand that I am not waiving any of my legal rights as a result of signing this consent form.
- I understand that there is no guarantee that this study will provide any benefits to me.

I understand that I am being asked to participate in a research study about whether a complex mental and social activities program or an exercise training program will assist in maintaining cognitive function. This study has been explained to me by _____________________.

Consent Continued

**Title: Reshaping the Path of Vascular Cognitive Impairment with Resistance Training**

The investigator and, when applicable, the substitute decision-maker (legally authorized representative) and are satisfied that the information contained in this consent form was explained to the participant to the extent that he/she is able to understand it, that all questions have been answered, and that the participant assents to participating in the research.

I will receive a signed copy of this consent form for my own records.

I consent to participate in this study.

# Signatures

_____________________________ ________________________ __________

Participant’s Signature Printed Name Date

_____________________________ ________________________ __________

Substitue Decision-maker’s Signature Printed Name Date

____________________________________ _________________________

Signature of Person Obtaining Consent Printed Name

___________________________________ _________________

Study Role Date
